# Optimizing Clinical Decision Making with Decision Curve Analysis: Insights for Clinical Investigators

**DOI:** 10.3390/healthcare11162244

**Published:** 2023-08-10

**Authors:** Daniele Piovani, Rozeta Sokou, Andreas G. Tsantes, Alfonso Stefano Vitello, Stefanos Bonovas

**Affiliations:** 1 Department of Biomedical Sciences, Humanitas University, 20072 Pieve Emanuele, Milan, Italy; 2 IRCCS Humanitas Research Hospital, 20089 Rozzano, Milan, Italy; 3 Neonatal Intensive Care Unit, “Agios Panteleimon” General Hospital of Nikea, Nikea, 18454 Piraeus, Greece; 4 Laboratory of Haematology and Blood Bank Unit, “Attiko” Hospital, School of Medicine, National and Kapodistrian University of Athens, 11527 Athens, Greece; 5 Microbiology Department, “Saint Savvas” Oncology Hospital, 11522 Athens, Greece; 6 Independent Researcher, 24123 Bergamo, Italy

**Keywords:** precision medicine, diagnosis, prognosis, clinical decision rules, models, statistical

## Abstract

A large number of prediction models are published with the objective of allowing personalized decision making for diagnostic or prognostic purposes. Conventional statistical measures of discrimination, calibration, or other measures of model performance are not well-suited for directly and clearly assessing the clinical value of scores or biomarkers. Decision curve analysis is an increasingly popular technique used to assess the clinical utility of a prognostic or diagnostic score/rule, or even of a biomarker. Clinical utility is expressed as the net benefit, which represents the net balance of patients’ benefits and harms and considers, implicitly, the consequences of clinical actions taken in response to a certain prediction score, rule, or biomarker. The net benefit is plotted against a range of possible exchange rates, representing the spectrum of possible patients’ and clinicians’ preferences. Decision curve analysis is a powerful tool for judging whether newly published or existing scores may truly benefit patients, and represents a significant advancement in improving transparent clinical decision making. This paper is meant to be an introduction to decision curve analysis and its interpretation for clinical investigators. Given the extensive advantages, we advocate applying decision curve analysis to all models intended for use in clinical practice.

## 1. Introduction

An increasing number of prediction models are being developed and validated in different fields of medicine [1]. Clinical prediction models provide an individualized prediction about prognosis or diagnosis. The probability of a future event of interest, or of an actual disease, may be estimated using biomarkers, clinical and imaging characteristics, or even genetic data and use a variety of statistical methods, from regressions to machine learning.

The ultimate goal of a prediction model should be to stratify patients based on their health prospects to assign the “right treatment to the right patient”. For example, to recommend an effective but risky treatment to high-risk patients. Alternatively, a prediction model may serve as a tool to refer a subset of patients to an expensive or inconvenient diagnostic test (e.g., biopsy).

Taking clinical action based on wrong predictions, however, may cause harm to patients. Statistical measures of discrimination, calibration, or other measures of model performance, while important [1], are not well-suited for directly and clearly assessing the clinical value of a score or biomarker due to a limited direct applicability to clinical practice [2]. Decision curve analysis (DCA) is an increasingly popular, valuable tool for judging whether a certain prediction model could be beneficial for patients. We believe clinicians should be aware of this method and know how to interpret its results.

## 2. Introducing Decision Curve Analysis: The Net Benefit

DCA was first developed by Vickers and colleagues in 2006 [3]. A free-text search in PubMed for “decision curve analysis” shows a dramatic surge in the popularity of this methodology over the last few years, with more than 3400 results retrieved only in 2022. Its use is recommended by the TRIPOD guidelines for developing prediction models [1]. DCA is a methodology to assess the clinical utility of a prognostic or diagnostic score/rule, or even a biomarker. Clinical utility is represented by the net benefit, which is defined by the following formula [4]:

Net benefit = (True positives/n) − (False positives/n) × (P_t_/(1 − P_t_))

where: n is the number of patients, and P_t_ (later referred to as P_threshold_) is the probability at the decision threshold, meaning the predicted probability of a certain outcome at which a clinician would decide to take appropriate action (e.g., to administer a treatment, perform an invasive diagnostic test, etc.). See the following sections.

Intuitively, a positive net benefit is desirable. Specifically, the net benefit can take values from minus infinity to a theoretical maximum which would coincide with the incidence of the outcome of interest for a perfectly accurate model (by definition less than 1.0) [3]. A perfect model would identify all patients who will develop the outcome (true positives), or have the disease in the case of a diagnostic model, and there would be no false positives. In this ideal case, the second term of the above formula would disappear, and the first remaining term would coincide with the outcome incidence in the target population.

Net benefit is directly interpretable on the scale of true positives. Differently from measures of discrimination and calibration, or any other measures of model performance, the interpretation of net benefit is straightforward. The net benefit is interpreted as the number of true positives found for every 100 patients in the target population, without regard to harm. Suppose we would like to interpret a net benefit of 0.10. This would mean that “*for every 100 patients in the target population, 10 true positives would be found without incurring harm*” (i.e., benefit is net). The adjective “net” is crucial in DCA because it indicates that the benefits are considered after subtracting the harms. The net benefit is similar to the concept of net balance in economics, where patient benefits can be assumed as revenues and patient harms as all the expenses. There will be more on this in the next sections. The net benefit can be also used to compare different clinical strategies.

### 2.1. The Exchange Rate

The exchange rate can be intuitively defined as the number of “false positives” that are worth one “true positive”. Numerically, it is the odds corresponding to the probability threshold (P_threshold_) over which a clinical action is taken, or below which an alternative clinical action is chosen. To understand what the exchange rate is, we have to define more clearly what the threshold probability (P_threshold_) is. The threshold probability refers to a specific probability value used in decision making. It represents the minimum or maximum probability at which a particular clinical action is deemed appropriate. For example, in diagnostics, a threshold probability may be set to determine whether a patient should be classified as having a specific condition or disease based on the probability of its presence.

We will also define individual predicted probability (P_predicted_) as the probability (e.g., of having the disease or of developing an outcome of interest) assigned to an individual patient, taking into account the relevant predictors or variables included in the prediction model. The individual P_predicted_ provides an estimate of the likelihood of a particular event occurring for that specific individual. A prediction model applied to a patient population should provide a broad spectrum of individual P_predicted_ reflecting the variability in health prospects/diagnosis of the sample population. Assuming a dichotomous outcome (e.g., diseased vs. non-diseased), an individual P_predicted_ will range from 0.0 to 1.0. For patients truly at low risk, the model will, hopefully, indicate a low or very low individual P_predicted_ (e.g., <5% or <10%), while for those truly at high risk the P_predicted_ will be closer to 1.0. Depending on the nature of the event and the consequences of a certain clinical action, a clinician would want to define a threshold for decision making, that is a threshold of P_predicted_ (i.e., P_threshold_) over which a clinical action should be undertaken.

### 2.2. The Core Problem of Clinical Decision Making: A Working Example

Let us consider a new hypothetical prognostic tool developed to evaluate the risk of distant metastases in patients with endometrial cancer 3 years after total hysterectomy. In low-risk endometrial cancer cases, surgery alone is considered sufficient for effective management. In high to intermediate risk endometrial cancer, adjuvant vaginal brachytherapy is recommended to maximize local control. This treatment option has relatively mild side effects. Conversely, in high-risk endometrial cancer patients, pelvic radiotherapy may be further added, particularly in stage I–II cases with risk factors; however, this increases the potential for side effects [5]. Hence, identifying patients who are at a higher risk of developing distant metastases is crucial for making informed treatment decisions, such as determining the need for adjuvant chemotherapy in combination with radiotherapy.

Let us simplify the clinical problem by focusing on the decision of administering adjuvant chemotherapy plus radiotherapy or not. This treatment option carries potential health benefits, such as reducing the risk of distant metastases, but also entails treatment-associated harms. The prediction model will always provide individual predicted probabilities ranging from 0.0 to 1.0. It is worth noting that some patients who would not develop distant metastases may still be recommended for treatment, which could potentially harm them.

To determine the plausible range of threshold probabilities (P_threshold_) for this decision, we can gather opinions from multiple oncologists. By asking the question, “*What probability of distant metastases at 3 years would you consider sufficient to refer a patient to adjuvant chemotherapy plus radiotherapy after hysterectomy?*”, we can explore different perspectives. Two extreme examples can be considered: the most “conservative” oncologist (oncologist 1) suggests a threshold value of 5%, while an oncologist highly concerned about side effects (oncologist 2) indicates a threshold of 50%. These examples serve to illustrate varying preferences.

The first clinician prioritizes preventing distant metastases and is willing to accept potential severe harms associated with the therapy by picking a low P_threshold_ which minimizes the chances to lose a true positive. A P_threshold_ of 5% can be interpreted as “*I am willing to treat 19 patients who would not develop distant metastases in order to treat one true positive*” (i.e., an exchange rate of 1:19). On the other hand, the second clinician, highly concerned about therapy side effects, opts for a threshold of 50% and is willing to treat only one patient who would not develop distant metastases in order to treat one true positive. The exchange rate in this case is the odds 1:1.

By considering these extreme examples, we aim to highlight the range of perspectives and individual trade-offs associated with treatment decisions. Please note that the exchange rates mentioned above are purely illustrative and serve to emphasize the different viewpoints.

### 2.3. How a Decision Curve Is Drawn, and How to Interpret It

**Figure 1:**
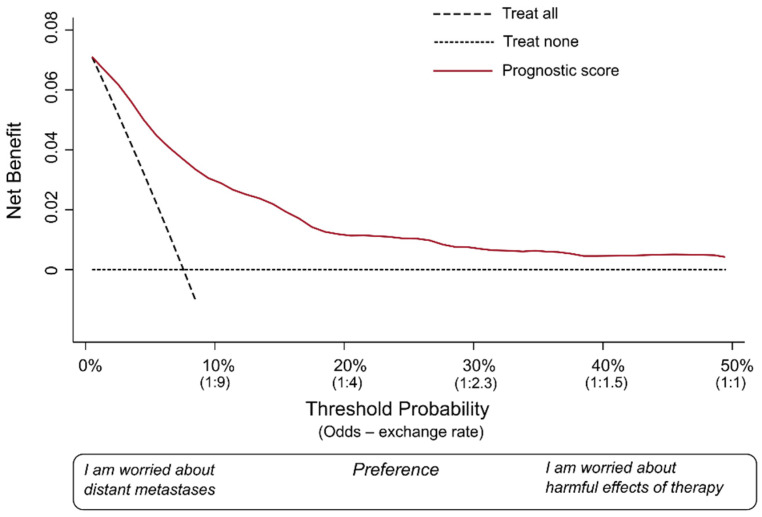
Hypothetical decision curve for a fictional prognostic model.

We covered the basic definitions of measures used in DCA. Let us draw a hypothetical decision curve for our fictional prognostic model (Figure 1).

The decision curve is a graphical representation that allows for the assessment of clinical strategies by evaluating their net benefit across different P_thresholds_. The x-axis represents the range of possible P_thresholds_, while the y-axis indicates the net benefit. The decision curve illustrates the trade-off between true-positive predictions and false-positive predictions for a given strategy. The area under the decision curve quantifies the overall clinical utility of the predictive model, capturing its ability to improve decision making compared to alternative approaches. In this hypothetical example, we considered that it would be very unlikely that an oncologist would consider P_thresholds_ > 50%. Most oncologists would probably recommend the therapy at much lower P_predicted_. Hence, we omitted to plot on the x-axis values over 50%. As suggested by Vickers and colleagues, we have also conveniently renamed the x-axis as “preference” [6], and reported the exchange rates corresponding to some meaningful P_threshold_.

In each DCA, there are at least two reference lines, one horizontal and one diagonal, depicting two possible alternative approaches against which the prediction model is compared. The dotted horizontal line indicates the net benefit of a strategy in which no patient is actually treated (i.e., treat none). The diagonal dashed line shows the net benefit of a clinical strategy in which all patients receive the therapy (i.e., treat all). These two lines represent the two most extreme strategies possible. In fact, any other clinical strategy would involve treating certain patients, and not treating others.

The red curve (i.e., the decision curve), instead, indicates the net benefit of a strategy in which all patients are scored by the illustrative prognostic model. This means each patient is assigned by the model a P_predicted_ of distant metastases, and the decision of whether to treat or not treat each patient is made according to the P_threshold_ the clinician has adopted. The net benefit is calculated for each possible P_threshold_ used for decision making. As the last step, the red curve is drawn by connecting all these points. Thus, this curve includes the extreme cases in our example (oncologists 1 and 2) and all other clinicians who may decide to choose a different P_threshold_.

Oncologist 1 was worried about the risk of metastases, had few concerns about the harmful effects of the therapy, and decided to use a P_threshold_ of 5% (i.e., patients with a P_predicted_ > 5% would be considered “positives” and would be recommended for treatment). As shown in Figure 1, in this fictional database of patients, a P_threshold_ of 5% would correspond roughly to a net benefit of 0.05. Oncologist 2 was more worried about the potentially severe harms of the particular therapy and decided to adopt a P_threshold_ of 50%. In this database of patients, the P_threshold_ would correspond roughly to a net benefit of 0.005. The important thing to note is that both are positive values.

The role of a DCA is to indicate the net benefit of taking clinical actions on the target population of patients over a large spectrum of possible P_thresholds_ and exchange rates, thus taking into account a variety of possible preferences. Just to reiterate, the term “preference” here means how many patients who would not develop metastases would be acceptable to treat, in order to also treat one patient who would truly develop metastases. In other words, adopting a lower P_threshold_ would favor sensitivity (i.e., the clinician is mostly worried about not missing true positives), while choosing a higher P_threshold_ would favor specificity by seeking to minimize false positives.

Figure 1 represents an ideal scenario of DCA. The net benefit at every single plausible P_threshold_ is higher than the net benefit offered by both strategies (“treat all”, “treat none”). The term “plausible” here means the range of possible exchange rates that would include the one adopted by the vast majority of the oncologists in this clinical scenario. We hypothesized that, in this example, this range could be from a P_threshold_ of 5% to 50%.

Let us briefly elaborate why this is important. In several clinical scenarios, “treat none” or “treat all” are real possibilities. In certain settings, a strategy may be to administer broad spectrum antibiotics as soon as possible to all patients with suspected sepsis (i.e., treat all). In case of a new controversial screening program for which there are not (yet) convincing proofs of effectiveness, the likely approach is to recommend it to nobody (i.e., treat none).

Though a prognostic score provides the same information to all clinicians, the P_threshold_ and the corresponding exchange rate that each clinician will adopt in practice may differ. This has to do with a variety of reasons, including personal professional experience, uncertainty and differences in interpreting the available evidence regarding a certain condition and its therapeutic options, but also patients’ preferences, including cost and convenience of possible alternatives. For example, which patients should be referred to a strategy of “extended close monitoring” for a certain chronic disease? In a low-income country where people have no access to universal insurance, the plausible P_threshold_ could be high (i.e., only patients showing high P_predicted_ would be advised to follow this strategy). Preference would likely differ in a Western country with a universal public health system. Hence, a new score should be, ideally, better than the “treat none” and “treat all” strategies over the entire spectrum of plausible preferences.

When more than one prognostic/diagnostic score is available, plotting net benefit of these different tools in the same graph makes it possible to directly compare the clinical utility of the new score over the existing one, or over other strategies that are well-established in clinical practice. To be adopted, a new score should offer some advantages. For example, a better net benefit at certain exchange rates, a similar net benefit while being more convenient to calculate, or a new diagnostic test which is less expensive or painful.

Interestingly, DCA is very useful also when developing a model. Fu et al. developed preliminary prediction models for intracranial infection in patients under external ventricular drainage and neurological intensive care by using three different approaches (i.e., logistic regression, support vector machines, and K-nearest neighbors) [7]. Although standard statistical measures of discrimination and calibration could not identify a clearly better modelling strategy, only the model developed using logistic regression had a positive net benefit over a large range of P_thresholds_ [7]. If a model net benefit is below zero at a certain P_threshold_, and all clinicians adopted that P_threshold_, not receiving the treatment would be a better solution for these patients. Under these circumstances, the model would make more harm than good. That is why we may not want to use in clinical practice a model whose net benefit is below zero for large intervals of plausible P_thresholds_. In case of serious miscalibration, this can happen even for models with apparently high AUC values (see next section) [7].

### 2.4. What Does DCA Add Compared to Measures of Discrimination and Calibration?

Prediction models have two fundamental features: discrimination and calibration [1]. In brief, commonly used measures of discrimination (e.g., the area under the receiver operating characteristic curve (AUC) and the c-statistic) quantify the ability of the model to identify patients with the disease/event of interest. An AUC or c-statistic equal to 0.50 would indicate a discrimination capacity not different than random chance (i.e., tossing a coin), whilst values closer to 1.0 would indicate an excellent discrimination capacity (i.e., patients who have the disease will be correctly identified).

Calibration, instead, is the agreement between the P_predicted_ and the observed frequency of event over the entire spectrum of predicted probabilities. Calibration is usually assessed graphically or with different statistical tests. When a prediction model constantly overestimates or underestimates the absolute probability of event, the clinical actions taken are ill-informed and may cause harm. This happens because the true absolute risk of an event/outcome is a key factor for decision making. Consider again the working example of this paper and a fictional P_threshold_ of 5% for suggesting adjuvant chemotherapy plus radiotherapy. Imagine dividing the P_predicted_ by the model by five times, hence creating a parallel imaginary model with high miscalibration. Due to how measures of discrimination are calculated, this would not affect the overall AUC of the model. Imagine, however, we tell a patient that the risk of relapse is 4% though it is really 20%. With this risk estimate, the patient would not be referred for treatment, hence missing an important opportunity to prevent distant metastases. This example makes clear that, even in cases of high AUC, the practical application of a miscalibrated prediction score may be harmful.

One, however, may wonder what DCA can add that is not already shown by measures of discrimination and calibration.

Consider a prediction model showing very high discrimination but some miscalibration. Hypothesize that the authors of this study would like to compare this new tool with a simpler existing tool which is used in practice and shows a worse discrimination but a better calibration. Which one is the best for patients? Choosing among the two tools based on conventional measures of discrimination and calibration would be rather subjective and arbitrary.

A practical example close to the above-mentioned scenario is the study by Perry and colleagues [8]. The authors were interested in developing a model for predicting up to 6-year risk of incident metabolic syndrome in young patients with psychosis from commonly recorded information [8]. At the developing phase they reached a model, the PsyMetRiC, which included some biochemical measurements that may not be widely available in this population. Hence, they also developed a simplified model not including such baseline variables. In the published paper, they showed that discrimination was rather comparable between the two models but there were some problems of miscalibration in the simplified model. By plotting the DCA curves of both models on the same graph, the authors showed how much the simplified model was comparable in terms of clinical utility and provided convincing evidence of the fact that both tools can serve the original purpose they had in mind without reaching a negative net benefit over a large spectrum of decision thresholds [8].

DCA goes beyond conventional measures of discrimination and calibration, as it considers them both at the same time [9], as well as individual preferences. DCA computes the net benefit over a spectrum of possible preferences. It allows for a comprehensive evaluation of the clinical utility of a model by considering the consequences of decisions made based on model predictions. Hence, plotting the results of DCA of both models in the same graph when applied on the same population of patients at risk would allow a direct comparison of the two prediction scores. This is why DCA is the tool of choice to assess which of the two decision tools would provide the highest (net) clinical utility in a given clinical scenario.

An important note here: DCA is a tool to help make informed decisions about which prediction model or approach may be most useful in clinical practice, but this does not mean that reporting the discrimination capacity and the calibration of any model is not necessary. On the contrary, they are fundamental measures that should always be reported for detailed transparency and comparability of published models [1]. DCA does not replace existing accuracy measures but complements them by considering the net benefit of a model in different clinical scenarios.

DCA offers valuable insights into the clinical utility of a prediction model in a specific patient population, but it cannot serve as a substitute for thorough external validation of a newly developed model in a different validation cohort. The assessment of external validity remains a crucial step in ensuring the generalizability of model performance to diverse patient populations and should always precede the widespread clinical application of any prediction model or clinical decision algorithm. DCA can be extended to the validation cohort to corroborate the sustained positive net benefit for patients within the new study population.

Another important disclaimer is that DCA is not a substitute for a traditional, comprehensive, decision analysis or cost-effectiveness analysis [6].

This paper is meant to be an introduction to DCA explaining the concepts in simple words with the help of examples. There are several additional analyses that can be performed with DCA, such as plotting the net reduction in interventions, or formally assigning the amount of—possibly different—harm corresponding to one or more diagnostic or treatment strategies (e.g., one or more imaging tests for the diagnosis of breast cancer). New developments of DCA are able to compare several treatment options when evidence comes from a network meta-analysis of clinical trials [10]. For additional information on what DCA can accomplish, we invite those interested to read further excellent papers on the topic [2,6,10,11].

## 3. Conclusions

Taking clinical action based on wrong predictions may cause harm to patients. Fundamental conventional measures of discrimination and calibration, while important, may not directly and clearly assess the clinical value of scores or biomarkers for diagnosis and prognosis.

DCA is a valuable and transparent tool displaying a measure of clinical utility, the net benefit, which takes into account discrimination and calibration at the same time, and is a measure of the net balance of patients’ benefits and harms. The net benefit considers, implicitly, the consequences of clinical actions taken in response to a certain prediction score, rule, or biomarker. It is plotted against an exchange rate, representing the spectrum of possible patients’ and clinicians’ preferences. This allows, for example, a comprehensive, comparative assessment of the clinical utility of different decision tools and alternative clinical strategies on a given population of patients.

Given the extensive advantages, we advocate applying DCA to all models intended for use in clinical practice, regardless of the computational method used for development (i.e., regression methods or machine learning approaches), as long as they provide an individual predicted probability.

As a closing remark, we would like to point out an important aspect. When a new prediction model is published, clinicians often inquire about the “optimal” P_threshold_ for decision making in a given scenario. In other terms, they seek “statistical” guidance on how to use the prediction model (i.e., at what predicted risk the patient should be recommended for treatment or referred to biopsy). We hope that this paper helps readers understand why this question cannot be answered through statistics alone [6]. Translating individualized predictions into clinical actions should always require both knowledge and careful judgement.

## Data Availability

No new data were created or analyzed in this study. Data sharing is not applicable to this article.

## References

[1] Moons K.G., Altman D.G., Reitsma J.B., Ioannidis J.P., Macaskill P., Steyerberg E.W., Vickers A.J., Ransohoff D.F., Collins G.S. (2015). Transparent Reporting of a Multivariable Prediction Model for Individual Prognosis or Diagnosis (TRIPOD): Explanation and Elaboration. Ann. Intern. Med..

[2] Halligan S., Altman D.G., Mallett S. (2015). Disadvantages of using the area under the receiver operating characteristic curve to assess imaging tests: A discussion and proposal for an alternative approach. Eur. Radiol..

[3] Vickers A.J., Elkin E.B. (2006). Decision Curve Analysis: A Novel Method for Evaluating Prediction Models. Med. Decis. Mak..

[4] Peirce C.S. (1884). The Numerical Measure of the Success of Predictions. Science.

[5] van den Heerik A.S.V.M., Horeweg N., de Boer S.M., Bosse T., Creutzberg C.L. (2021). Adjuvant Therapy for Endometrial Cancer in the Era of Molecular Classification: Radiotherapy, Chemoradiation and Novel Targets for Therapy. Int. J. Gynecol. Cancer.

[6] Vickers A.J., van Calster B., Steyerberg E.W. (2019). A Simple, Step-by-Step Guide to Interpreting Decision Curve Analysis. Diagn. Progn. Res..

[7] Fu P., Zhang Y., Zhang J., Hu J., Sun Y. (2022). Prediction of Intracranial Infection in Patients under External Ventricular Drainage and Neurological Intensive Care: A Multicenter Retrospective Cohort Study. J. Clin. Med..

[8] Perry B.I., Osimo E.F., Upthegrove R., Mallikarjun P.K., Yorke J., Stochl J., Perez J., Zammit S., Howes O., Jones P.B. (2021). Development and external validation of the Psychosis Metabolic Risk Calculator (PsyMetRiC): A cardiometabolic risk prediction algorithm for young people with psychosis. Lancet Psychiatry.

[9] Van Calster B., Vickers A.J. (2015). Calibration of risk prediction models: Impact on decision-analytic performance. Med. Decis. Mak..

[10] Chalkou K., Vickers A.J., Pellegrini F., Manca A., Salanti G. (2023). Decision Curve Analysis for Personalized Treatment Choice between Multiple Options. Med. Decis. Mak..

[11] Vickers A.J., Van Calster B., Steyerberg E.W. (2016). Net Benefit Approaches to the Evaluation of Prediction Models, Molecular Markers, and Diagnostic Tests. BMJ.

